# Selected Papers from the 1st International Online Conference on Nanomaterials

**DOI:** 10.3390/nano9071021

**Published:** 2019-07-17

**Authors:** Ana María Díez-Pascual, Guanying Chen

**Affiliations:** 1 Department of Analytical Chemistry, Physical Chemistry and Chemical Engineering, Faculty of Sciences, University of Alcalá, 28805 Alcalá de Henares, Madrid, Spain; 2 School of Chemistry and Chemical Engineering, Harbin Institute of Technology, Harbin 150001, China

After decades of intense research, nanomaterials are now an integral part of many applications and enjoy the attention of a large research community. Intrinsically multidisciplinary, research activities are spanning from engineering, over physics and chemistry, to biology and medicine. Nanomaterials, as the name indicates, are extremely tiny, less than a millionth of a meter in size. They encompass exceptional physical and chemical features which provide them enhanced properties compared to their macroscopic counterparts, such as higher reactivity and strength, superior thermal and electrical characteristics as well as functionality. These advantages have led to nanomaterials being included in a broad range of consumer products. The transport, electronics, cosmetics, healthcare, and sport industries all benefit from nanotechnology advances. Novel fields have also appeared, such as nanomedicine, which plans to drastically change our future ability to treat disease.

This Special Issue compiles five selected papers from the Proceedings of the 1st International Online Conference on Nanomaterials, held 1–15 September 2018 on sciforum.net, an online platform for hosting scholarly e-conferences and discussion groups. It targets a broad readership of physicists, chemists, materials scientists, biologists, environmentalists, and nanotechnologists, and provides interesting examples of the most recent advances in the synthesis, characterization, and applications of nanomaterials. The papers present very different types of nanomaterials, such as double hydrophilic branched copolymers of poly(N,N-dimethyl acrylamide) and poly(ethylene oxide) [1], carbon nano-dots (CNDs)/poly(vinyl alcohol) (PVA) nanocomposites [2], polyacrylamide/SiO_2_ hydrogel nanocomposites [3], silicon quantum dots (Si QDs) and iron oxide (α-Fe_2_O_3_) nanoparticles [4], and hexamethylene diisocyanate (HDI)-functionalized graphene oxide (GO) [5]. In the paragraphs that follow, a concise overview of each of the published articles is provided in order to attract the interest of potential readers.

Recently, hydrogels have emerged as ideal candidates for numerous biomedical and biotechnological applications due to their unique physical and biochemical properties [6]. They comprise very porous and hydrated networks that enable cell encapsulation for tissue engineering, the loading and release of bioactive molecules for drug delivery, and wound dressing and biosensing applications [7]. Nonetheless, their poor mechanical performance limits their use in certain applications, and strong effort has been carried out on improving their properties via incorporation of nanofillers such as inorganic nanoparticles [8], though the precise mechanisms behind such enhancements are not fully understood yet. To get more insight into the role of nanoparticles on the mechanical properties of hydrogel nanocomposites, Zaragoza et al. [3] synthesized chemically crosslinked polyacrylamide hydrogels reinforced with SiO_2_ nanoparticles as a model system of study. Rheological experiments revealed that the improvements induced by means of the nanoparticles surpass the maximum modulus that can be attained via simply chemical crosslinking. Furthermore, results demonstrate that the concentrations of the nanoparticle, monomer, and chemical crosslinker play a key role in mechanical improvement, which is crucial for their use in a wide range of applications.

Carbon nano-dots (CNDs) represent a novel class of carbon-based nanomaterials with quasi-spherical shape and ultra-small size of ~10 nm, that has attracted a lot of attention among studies, owed to their valuable characteristics such as cheapness, biodegradability, strong and broad optical absorption, and high chemical stability [9]. These extraordinary properties make them useful for a variety of fields including biosensing, bioimaging, drug delivery, optoelectronics, photovoltaics, and photocatalysis. In particular, the rich optical and electronic properties of CNDs including efficient light harvesting, tunable photoluminescence, and superior photoinduced electron transfer have involved considerable interest in different photocatalytic applications. The addition of CNDs to polymeric matrices is currently under intense study since they display great potential for light emitting diodes (LEDs), flexible electronic displays, and other optoelectronic applications [10]. In this context, Aziz et al. [2] investigated the effect of CNDs on the UV absorption spectra of PVA-based nanocomposites prepared via solution casting. The existence of CNDs of different sizes well dispersed throughout the matrix was proved via microscopic observations. In addition, the gradual increase of the refractive index with increasing CND concentration corroborated the homogeneous distribution of the carbon nanofillers all over the host PVA. The Infrared spectra and X-ray diffraction data demonstrated the complex formation between PVA and CNDs. Further, the CNDs caused strong absorptions at 280 and 330 nm assigned to n-π* and π-π* transitions, as well as a reduction in the optical bandgap.

Another highly interesting carbon-based nanomaterial is graphene oxide (GO), the oxidized form of graphene, which exhibits exceptional properties including high mechanical strength, optical transparency, amphiphilicity, and surface functionalization capability [11]. Even though, its insolubility in non-polar and polar aprotic solvents limits certain applications. To work out this matter, new functionalization approaches are required [12]. In this regard, Luceño-Sanchez et al. [5] prepared and characterized a series of hexamethylene diisocyanate (HDI)-functionalized GO. Several reaction conditions were tested to maximize the degree of modification, and comprehensive characterizations were carried out by means of elemental analysis, Infrared, and Raman spectroscopies to verify the accomplishment of the functionalization reaction. The surface morphology of the modified samples was explored by microscopic techniques, which showed a rise in the sheet thickness with increasing the level of modification. The HDI-GO was found to be more hydrophobic in nature than neat GO and was easily suspended in polar aprotic solvents such as N,N-dimethylformamide (DMF), N-methylpyrrolidone (NMP) and dimethyl sulfoxide (DMSO), as well as in low polar/non-polar solvents like tetrahydrofuran (THF), chloroform and toluene. Further, the dispersibility increased with increasing degree of modification. Besides, it was found that the covalent bonding of HDI enhances the thermal stability of GO due to chemical crosslinking between neighboring sheets, which is advantageous for long-term electronics and electrothermal device applications. These HDI-GO samples are perfect candidates as nanofillers for the development of high-performance GO-based polymeric nanocomposites [13,14].

The use of nanomaterials as optical sensors is emerging as a new frontier in nanoscience, which enables to detect hint analyte of varying kinds (temperature, oxygen molecules, peroxide, disease biomarkers, etc.) at high precision [15]. Moreover, nanomaterials optical sensing can empower remote and noninvasive evaluation of microenvironment at a nanometric resolution, providing unprecedented opportunities for quantifying analytes of interest, such as humidity [16]. Humidity is present everywhere and is one of the most important physical parameters in semiconductors, electronics, food processing, and pharmaceutical industries wherever the quality of products is influenced by water molecules. In this context, Lazarova et al. described a thin film humidity optical sensor with nanometric thicknesses, 100–400 nm, prepared from double hydrophilic copolymers of complex branched structures (containing poly(N,N-dimethyl acrylamide) and poly(ethylene oxide) blocks) [1]. The polymer thin films were deposited on top-covered Bragg stacks with a sputtered Au–Pd film (30 nm) that bring color for the colorless polymer/glass system, thus enabling transmittance measurements for humidity sensing. The humidity content was quantitatively studied by calculating the color coordinate alternation using the measured spectra of transmittance. This work provides a paradigm of using top covered Bragg stacks and polymer/metal thin film structures as sensitive humidity optical sensors.

Nanomedicine represents the next era for personalized disease prevention and treatment with better performance and fewer side effects. One important direction of nanomedicine involves the use of nanocarriers to deliver drugs or prodrugs to intended sites in a stimuli-controlled manner for precise theranostic treatment and actuation in a living organism [17]. Among various delivery ways, oral drug administration is still the preferred route in this regard, with advantages including patient comfort, reduced chances of infection, and minimal invasiveness. However, orally administered drugs usually have to be assimilated via the gastrointestinal (GI) tract, which then crosses the small intestinal barrier to reach vascular circulation for intended sites [18]. As such, an in vitro model to assess intestinal absorption is of paramount importance to mimic the conditions of drug-loaded nanocarriers in GI. To do so, Strugari et al. co-cultured Caco-2 (cat. no. CRL-2102) cells and human adenocarcinoma line HT-29 preconditioned in methotrexate (MTX) on a Transwell^®^ systems in a monolayer [4]. This co-culture model allows for modulating intercellular junction geometry, thereby fine-tuning the effective permeability of the monolayer by simply adjusting the initial cell seeding ratio of Caco-2/HT29-MTX (7:3 and 5:5 in the work). The monolayer integrity is assessed by measuring transepithelial electrical resistance (TEER) using chopstick electrodes inserted in the apical (AP) and basolateral (BL) compartments of the well. This model was exposed to non-cytotoxic concentration levels (20 μg/mL) of Si QDs and α-Fe_2_O_3_ nanoparticles, quantitatively evaluating their ability to penetrate through the intestinal mucus barrier, and their influence on the morphological alterations of the Caco-2/HT29-MTX. However, the obtained results showed that the current surface of these nanoparticle suspensions prevented them from diffusing across the intestinal model, demonstrating the importance of nanoparticle surface functionalization to enable them to cross the intestinal barriers.

These papers published in the special issue of the 1st International Online Conference on Nanomaterials (IOCON) showcase the important roles of nanomaterials to influence science and technology and human healthcare. We hope that more and more scientists can join the open-access ICON forum in the future to facilitate the advancement of nanoscience together.
